# Supplementary material for: Petal abscission is promoted by jasmonic acid-induced autophagy at Arabidopsis petal bases
Source: Nat Commun. 2024 Feb 6;15:1098. doi: 10.1038/s41467-024-45371-3 (PMC10847506; doi:10.1038/s41467-024-45371-3)
Supplement: Supplementary file 1 — Supplementary Information [file 41467_2024_45371_MOESM1_ESM.pdf]

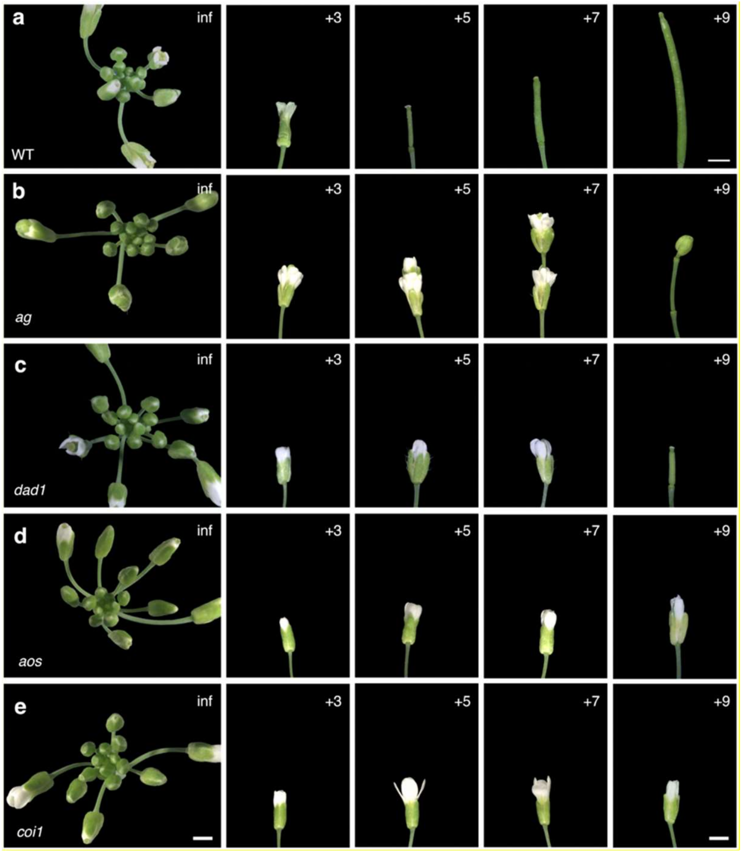

Supplementary Fig. 1 Close-up views of flowers from JA-related mutants during petal abscission.

a-e, Left, Top views of wild-type (WT, Col-0), *ag*, *dad1*, *aos*, and *coi1* inflorescences. Right, Side views of WT, *ag*, *dad1*, *aos*, and *coi1* flowers at the indicated positions. Scale bars = 1 cm.

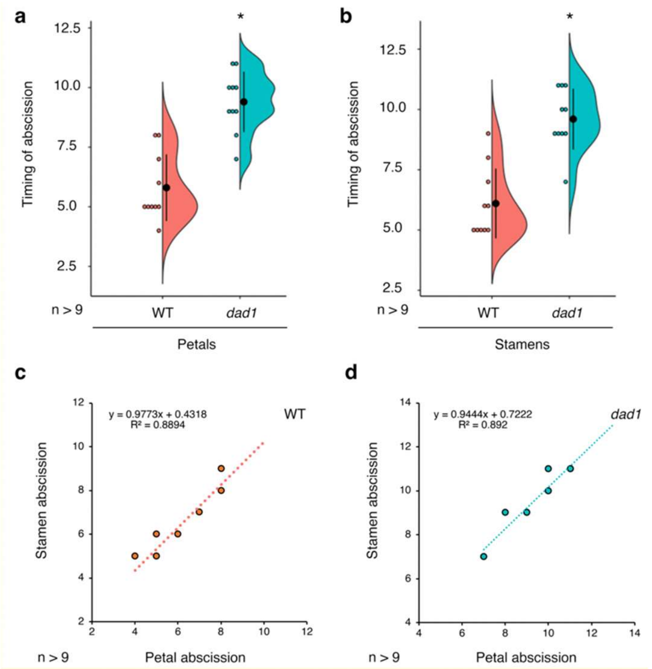

Supplementary Fig. 2 Petal and stamen abscission in WT and *dad1*.

a, **b**, Timing of abscission of petals (a) and stamens (b), shown as individual data points (left) and violin plots (right) for each genotype. Black dots and vertical lines indicate mean and standard deviation (SD), respectively. $n > 9$. Asterisks indicate significant differences between the WT and *dad1* based on two-tailed Student's *t*-test. **c, d**, Scatterplots between timing of petal and stamen abscission in the WT (c) and *dad1* (d). The Pearson's correlation coefficients are shown.

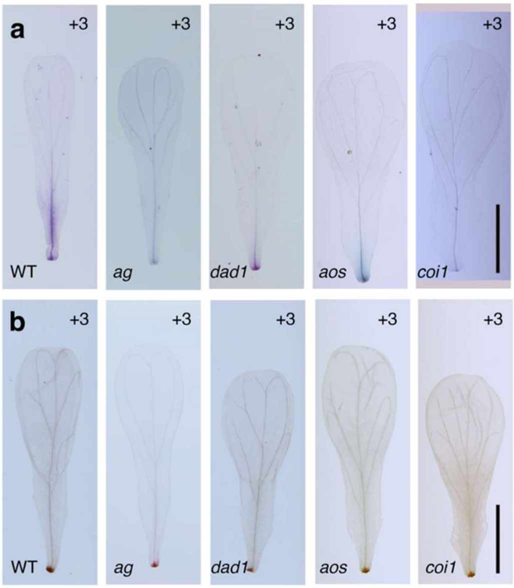

Supplementary Fig. 3 Entire petal images of programmed cell death and H_2O_2 accumulation during petal abscission.

a, Trypan blue staining of WT, *ag*, *dad1*, *aos*, and *coi1* petals from position –3 to +4 flowers. Scale bar =1 mm. **b**, DAB staining of WT, *ag*, *dad1*, *aos*, and *coi1* petals from position –3 to +4 flowers. Scale bar = 1 mm.

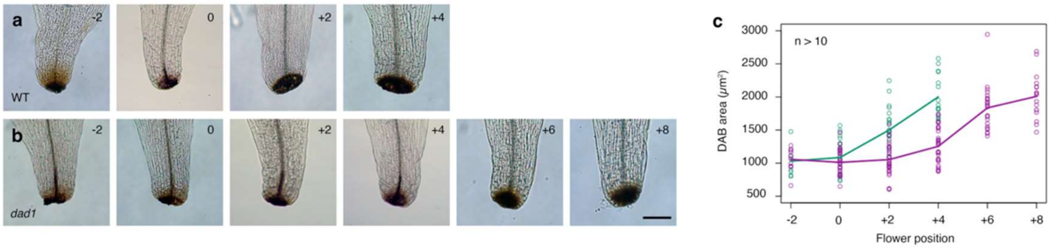

Supplementary Fig. 4 DAB staining pattern in petals during petal abscission.
a, **b**, Time course of DAB staining for WT (a) and *dad1* (b) petals from position –2 to +4 flowers. Scale bar = 100 µm. **c,** Quantification of DAB-stained area (µm^2). Area averages in WT and *dad1* are shown with green and purple lines, respectively, with individual data points.

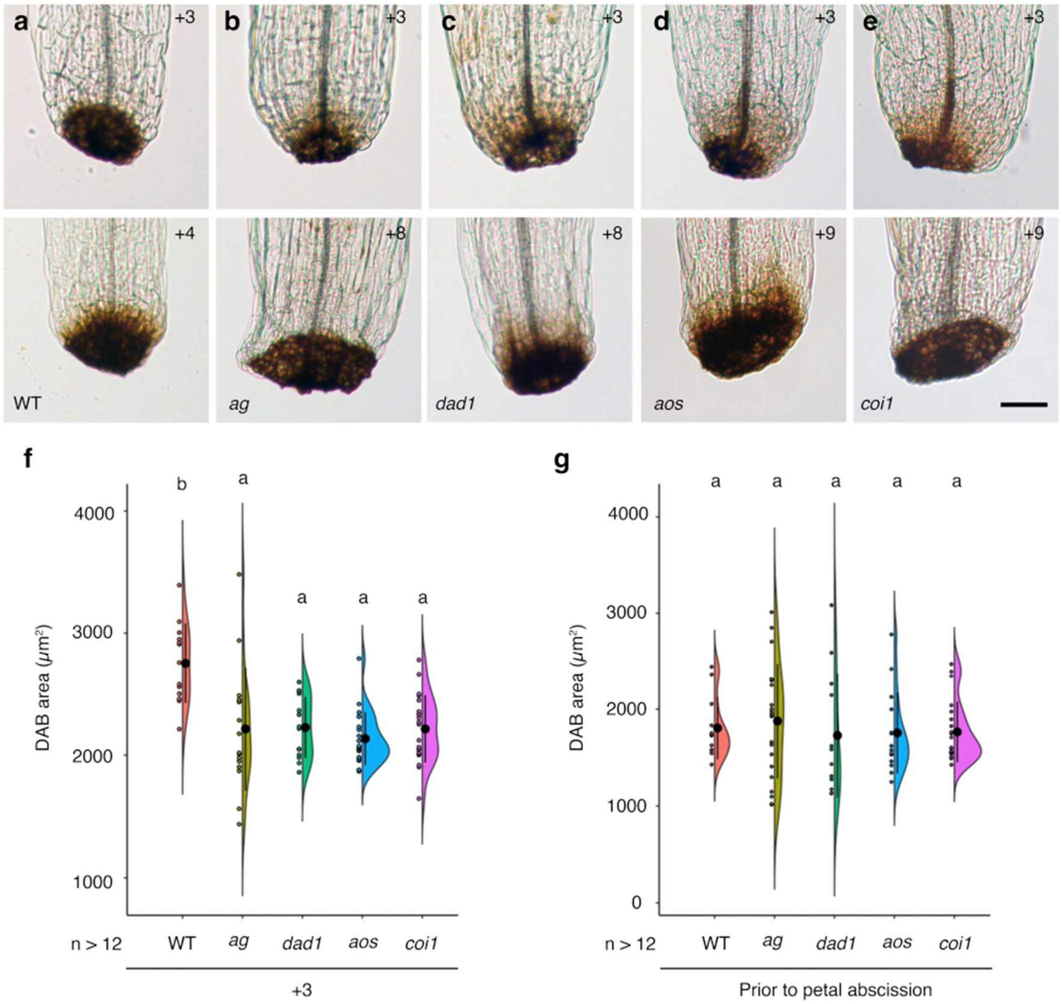

Supplementary Fig. 5 DAB staining of petals at two different positions during petal abscission.

a-e, DAB staining in petals of WT (a), *ag* (b), *dad1* (c), *aos* (d), and *coi1* (e) at position +3 (above) and just before petal abscission (below). Scale bar = 50 μm. **f, g,** Quantification of DAB-stained area (μm^2), shown as individual data points (left) and violin plots (right) for each genotype. Position +3 (f) and just before petal abscission (g) are shown. Black dots and vertical lines indicate mean and SD, respectively. $n > 10$. Different letters indicate significant differences, based on one-way ANOVA and post-hoc Tukey's HSD test ($p < 0.05$).

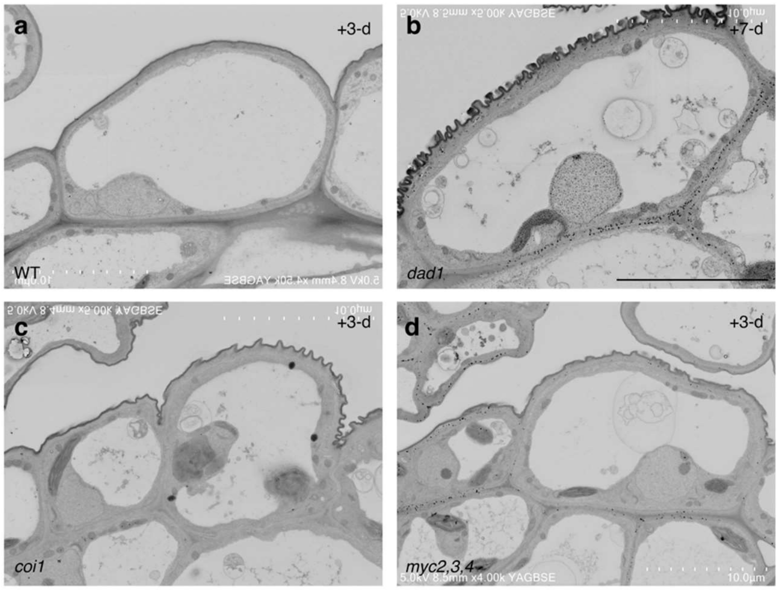

Supplementary Fig. 6 Observation of vacuole size during petal abscission.
a-d, SEM images of position d cells from position +3 petals in the WT (a), position +7 petals in *dad1* (b), position 3 petals in *col1* (c), and position +3 petals in *myc2 myc3 myc4* (*myc2*,*3*,*4*, d). Scale bar = 10 µm.

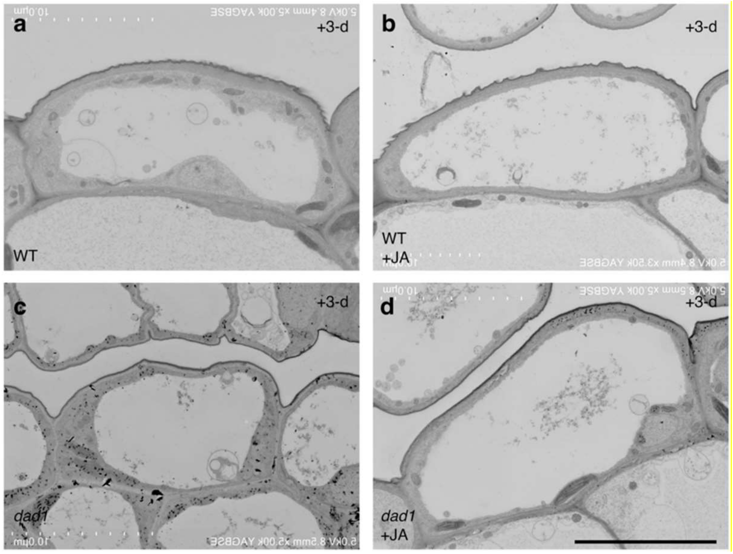

Supplementary Fig. 7 Rescue of vacuole size in *dad1* by JA treatment.
a-d, SEM images of position d cells from position 3 petals in mock- (a) and JA-treated (b) WT, and mock- (c) and JA-treated (d) *dad1*. Scale bar = 10 µm.

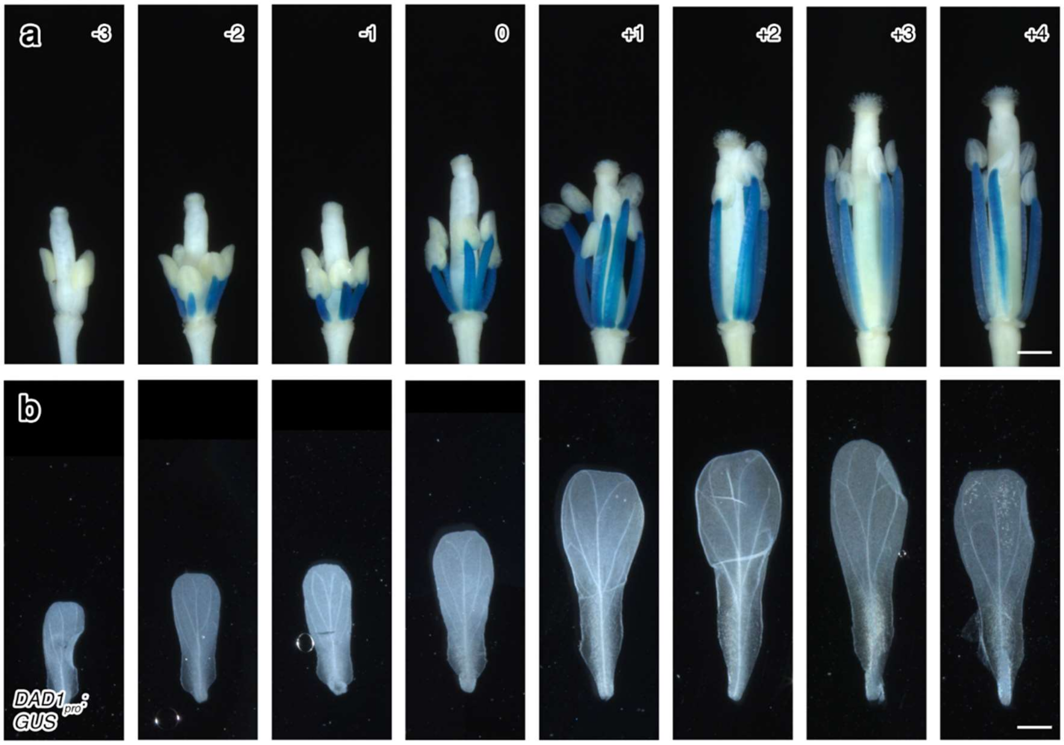

Supplementary Fig. 8 Expression domain of *DAD1$_{pro}$:GUS* during petal abscission.
a, *DAD1$_{pro}$:GUS* staining pattern in WT stamens from position –3 to +4 flowers. Scale bar = 500 µm. **b,** *DAD1$_{pro}$:GUS* staining pattern in WT petals (b) from position –3 to +4 flowers. Scale bar = 500 µm.

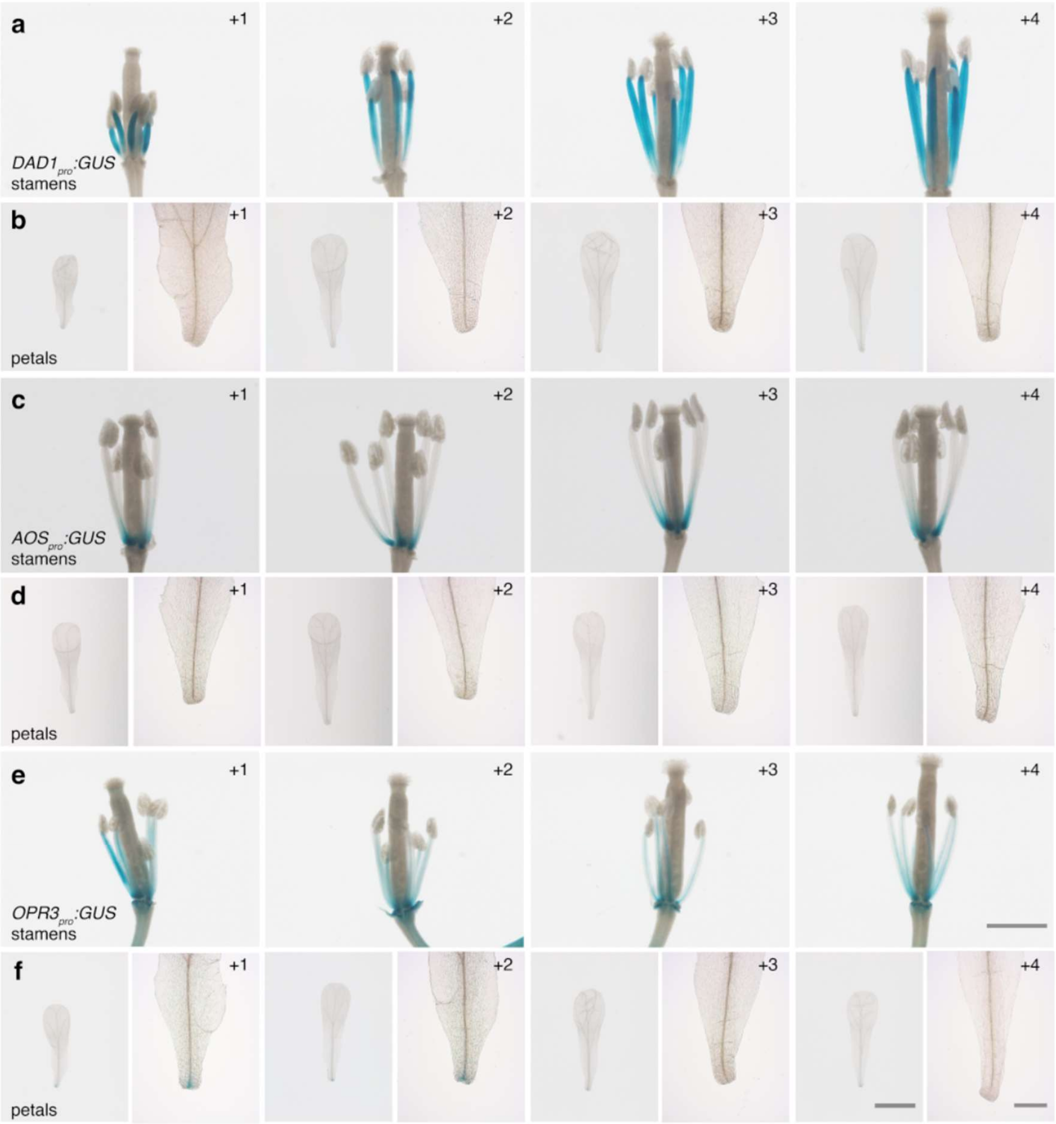

Supplementary Fig. 9 Expression domains of JA biosynthesis genes during petal abscission.

a, *DAD1pro:GUS* staining pattern in WT stamens in position +1 to +4 flowers. **b,** *DAD1pro:GUS* staining pattern in WT petals (b) in position +1 to +4 flowers. **c,** *AOSpro:GUS* staining pattern in WT stamens in position +1 to +4 flowers. **d,** *AOSpro:GUS* staining pattern in WT petals (b) in position +1 to +4 flowers. **e,** *OPR3pro:GUS* staining pattern in WT stamens in position +1 to +4 flowers. **f,** *OPR3pro:GUS* staining pattern in WT petals (b) in position +1 to +4 flowers. Scale bars = 500 μm.

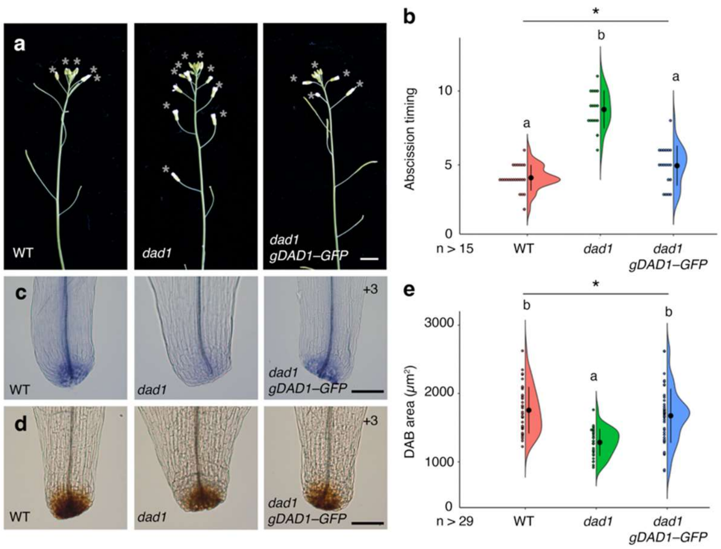

Supplementary Fig. 10 Rescue of the *dad1* mutant by introduction of *gDAD1–GFP*.

a, Profile view of WT, *dad1*, and *dad1 gDAD1–GFP* inflorescences. Opened flowers with petals are indicated by gray asterisks. Scale bar = 1 cm. **b,** Quantification of abscission timing, shown as individual data points (left) and violin plots (right) for each genotype. Black dots and vertical lines indicate mean and standard deviation, respectively. $n > 15$. Different letters indicate significant differences, based on one-way ANOVA and post-hoc Tukey's HSD test ($p < 0.05$). **c,** Trypan blue staining of WT, *dad1*, and *dad1 gDAD1–GFP* petals from position +3 flowers. Scale bar =100 µm. **d,** DAB staining of WT, *dad1*, and *dad1 gDAD1–GFP* petals from position +3 flowers. Scale bar = 100 µm. **e,** Quantification of DAB-stained area (µm^2), shown as individual data points (left) and violin plots (right) for each genotype. Black dots and vertical lines indicate mean and standard deviation, respectively. $n > 29$. Different letters indicate significant differences, based on one-way ANOVA and post-hoc Tukey's HSD test ($p < 0.05$).

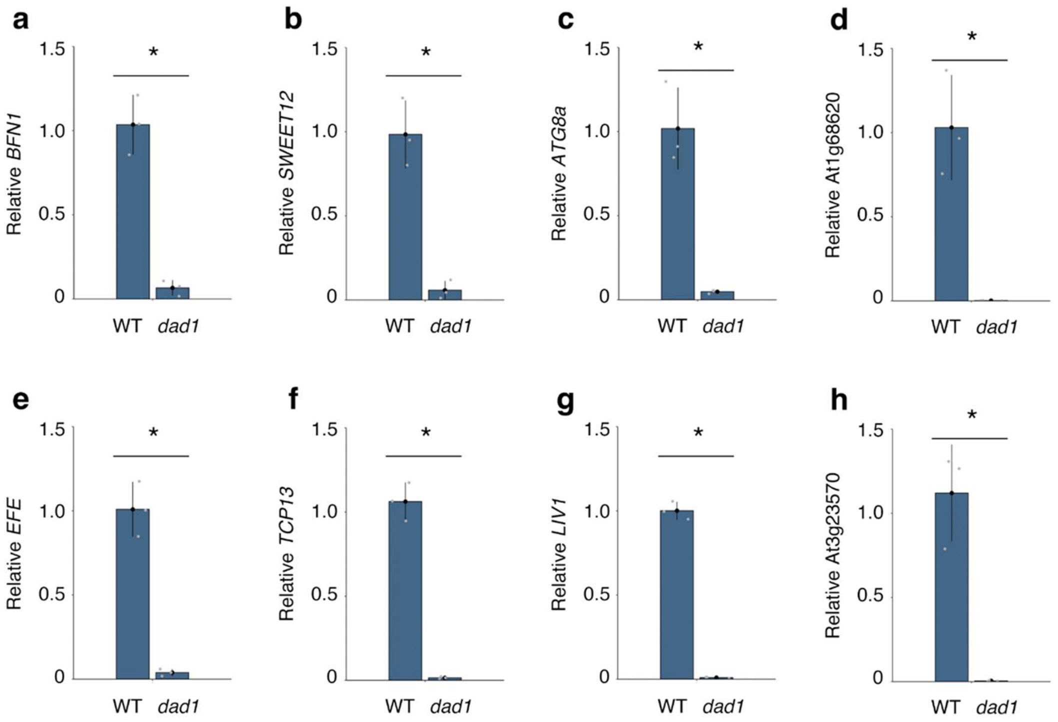

Supplementary Fig. 11 Expression levels of selected DEGs in WT and the *dad1* mutant. **a-h**, Gene expression levels in WT and the *dad1* mutant, as determined by RT–qPCR. a, *BFN1*. b, *SWEET12*. c, *ATG8a*, d, At1g68620. e, *EFE*. f, *TCP13*. g, *LIV1*. h. At3g23570. Data are means ± standard error of the mean (SEM). *n* = 3. Asterisks indicate significant differences between WT and *dad1* based on two-tailed Student's *t*-test.

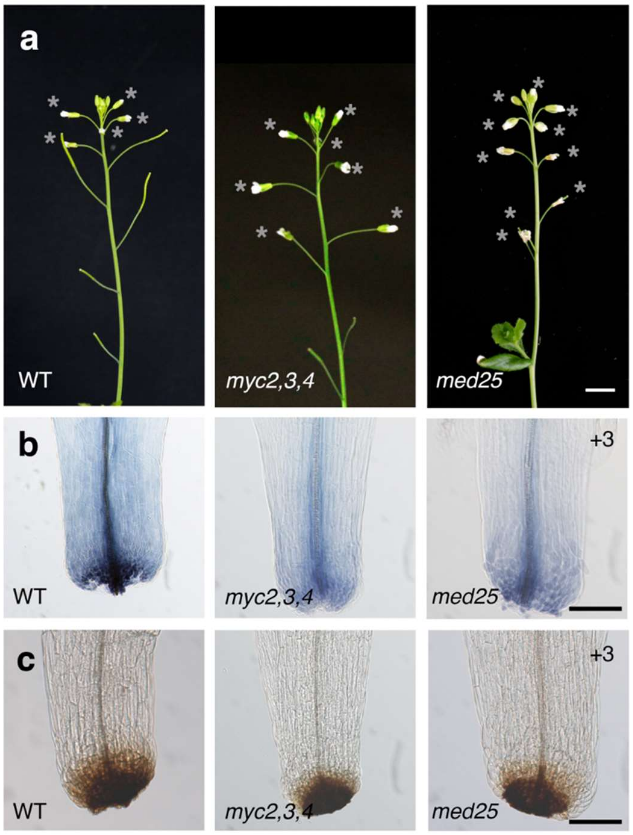

Supplementary Fig. 12 Petal abscission in the *myc2 myc3 myc4* and *med25* mutants.
a, Profile view of WT, *myc2 myc3 myc4* (*myc2*,*3*,*4*), and *med25* inflorescences. Opened flowers with petals are indicated by asterisks. Scale bar = 1 cm. **b,** Trypan blue staining of WT, *myc2 myc3 myc4*, and *med25* petals at position 3 flowers. Scale bar = 100 µm. **c,** DAB staining of WT, *myc2 myc3 myc4*, and *med25* petals at position 3 flowers. Scale bar = 100 µm.

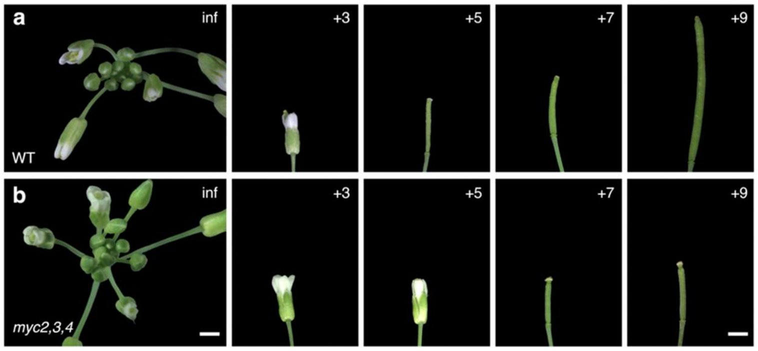

Supplementary Fig. 13 Close-up views of *myc2 myc3 myc4* flowers during petal abscission.

a, b, Left, Top views of WT (a), and *myc2 myc3 myc4* inflorescences. Right, Side views of WT, and *myc2 myc3 myc4* flowers at the indicated positions. Scale bar = 1 cm.

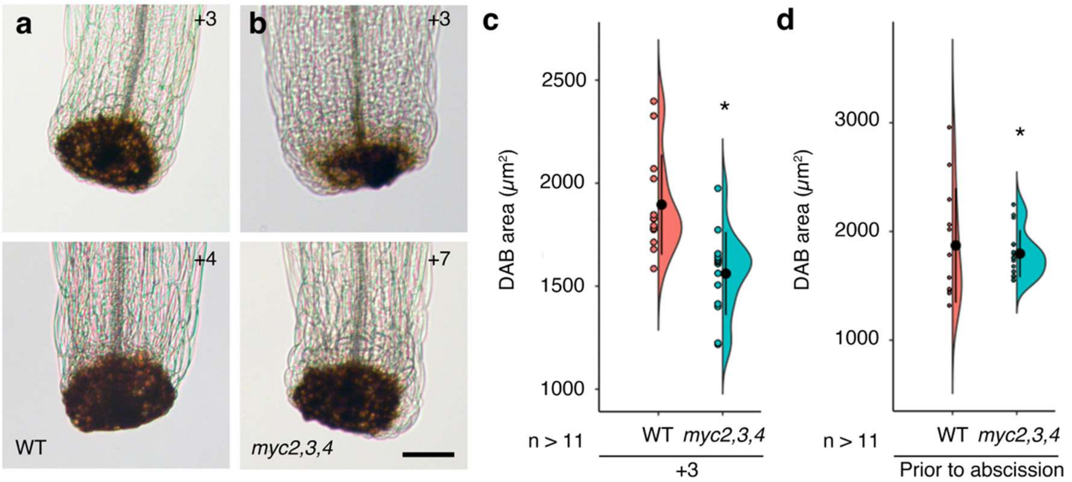

Supplementary Fig. 14 DAB staining in petals from flowers at two different positions during petal abscission.

a, b, DAB staining in petals of WT (a), and *myc2 myc3 myc4* (b) flowers at position +3 (above) and just before petal abscission (below). Scale bar = 50 µm. **c, d,** Quantification of DAB-stained area (µm^2), shown as individual data points (left) and violin plots (right) for each genotype. Position +3 (c) and just before petal abscission (d) are shown. Black dots and vertical lines indicate mean and SD, respectively. $n > 11$. Asterisks indicate significant differences between WT and *myc2 myc3 myc4* based on two-tailed Student's *t*-test.

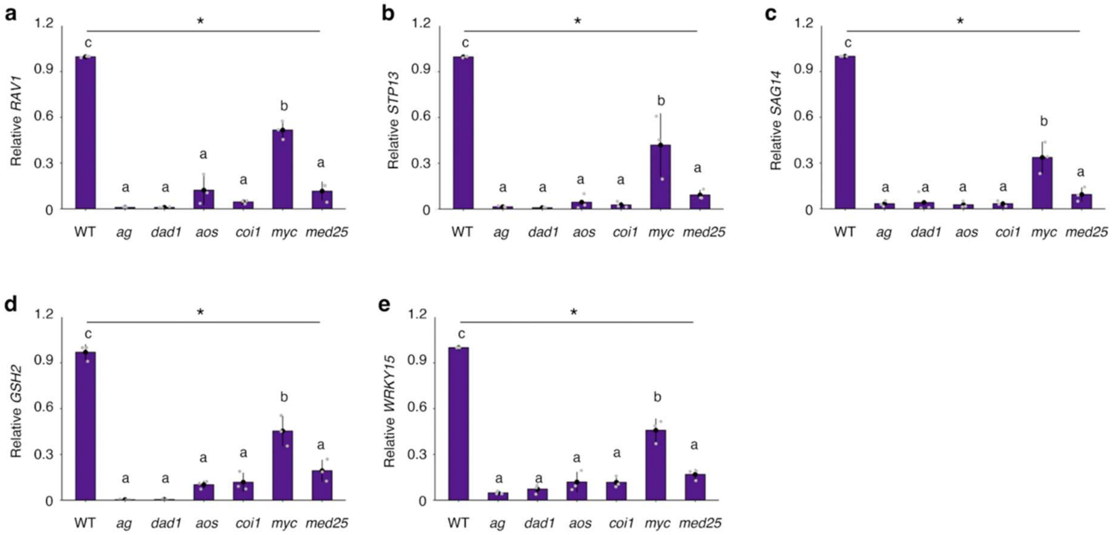

Supplementary Fig. 15 Expression levels of shared direct targets of MYC2 and MED25 in WT and JA-defective mutants.

Relative expression levels of the indicated genes in WT and JA-defective mutants as determined by RT–qPCR. a, *RAV1*. b, *STP13*. c, *SAG14*, d, *GSH2*. e, *WRKY15*. Results are from three independent experiments. Data are means ± SEM. $n = 3$. Different letters indicate significant differences, based on one-way ANOVA and post-hoc Tukey's HSD test ($p < 0.05$).

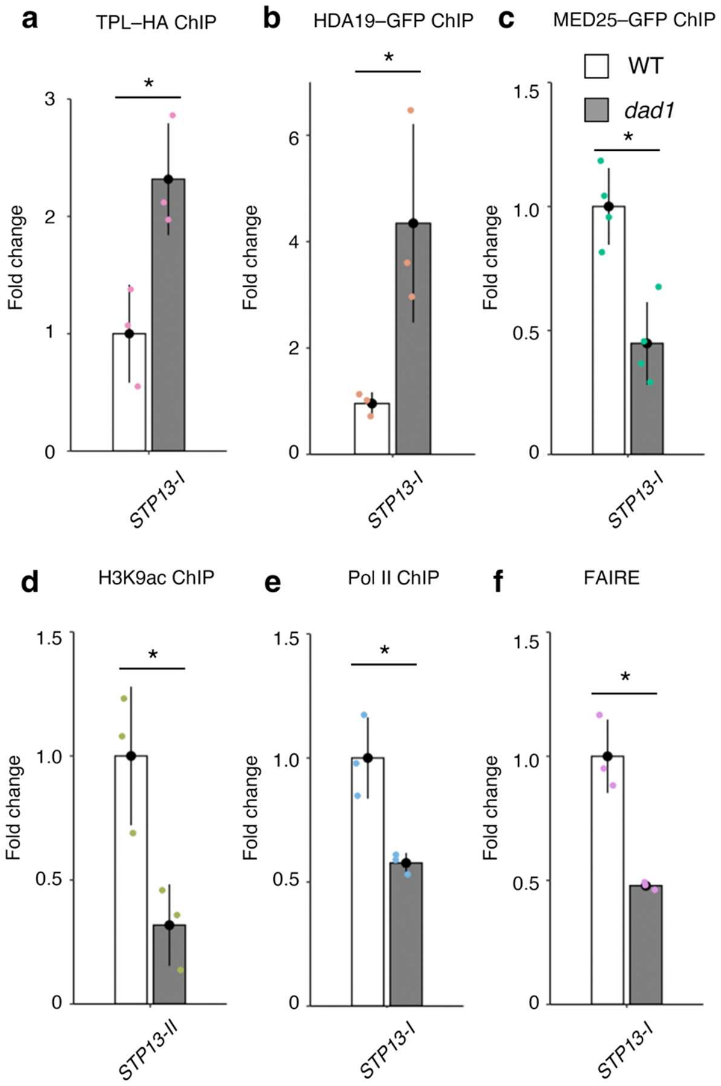

Supplementary Fig. 16 JA-mediated chromatin state switch at the *STP13* promoter during petal abscission.

a-e, Binding of TPL–HA (a), HDA19–GFP (b), MED25–GFP (c), H3K9ac (d), and Pol II (e) at the *STP13* promoter in WT and the *dad1* mutant, as determined by ChIP–qPCR. Positions of PCR amplicons for ChIP–qPCR are shown in Fig. 4c. Data are means ± SEM. $n = 3$. Asterisks indicate significant differences between WT and *dad1* based on two-tailed Student's *t*-test. **f**, Chromatin accessibility at the *STP13* promoter in WT and the *dad1* mutant by FAIRE–qPCR. Positions of PCR amplicons for ChIP–qPCR are shown in Fig. 4c. Data are means ± SEM. $n = 3$. Asterisks indicate significant differences between WT and the *dad1* mutant based on two-tailed Student's *t*-test.

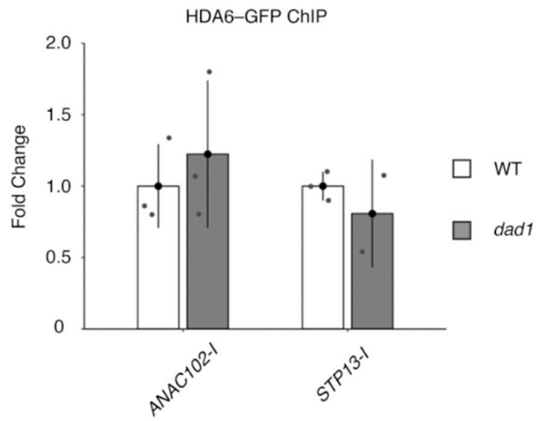

Supplementary Fig. 17 Binding of HDA6–GFP to the *ANAC102* and *STP13* promoters. Binding of HDA6–GFP at the *ANAC102* promoter in WT as shown by ChIP–qPCR. Positions of PCR amplicons for ChIP–qPCR are shown in Fig. 4c. Data are means ± SEM. n = 3. No significant differences between WT and the *dad1* mutant were observed based on two-tailed Student's t-test.

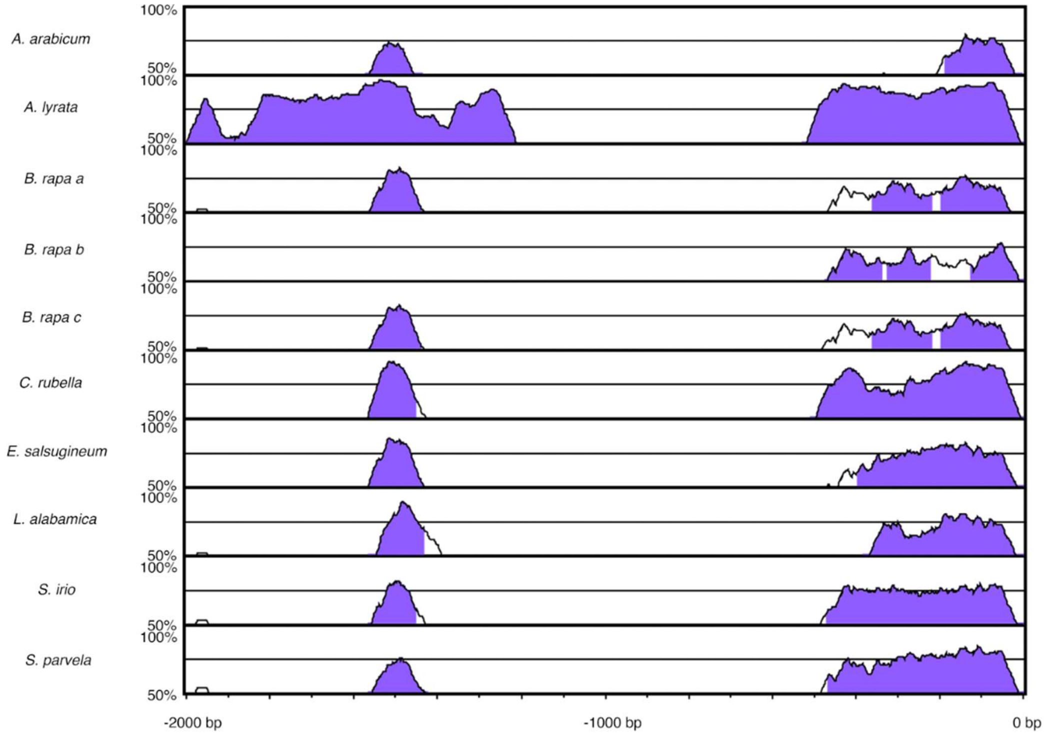

Supplementary Fig. 18 Phylogenetic shadowing across nine *Brassicaceae* species. Pairwise alignment created by mVISTA of the 5’ upstream intergenic region of the *ANAC102* promoter. +1 bp corresponds to the translation start site.

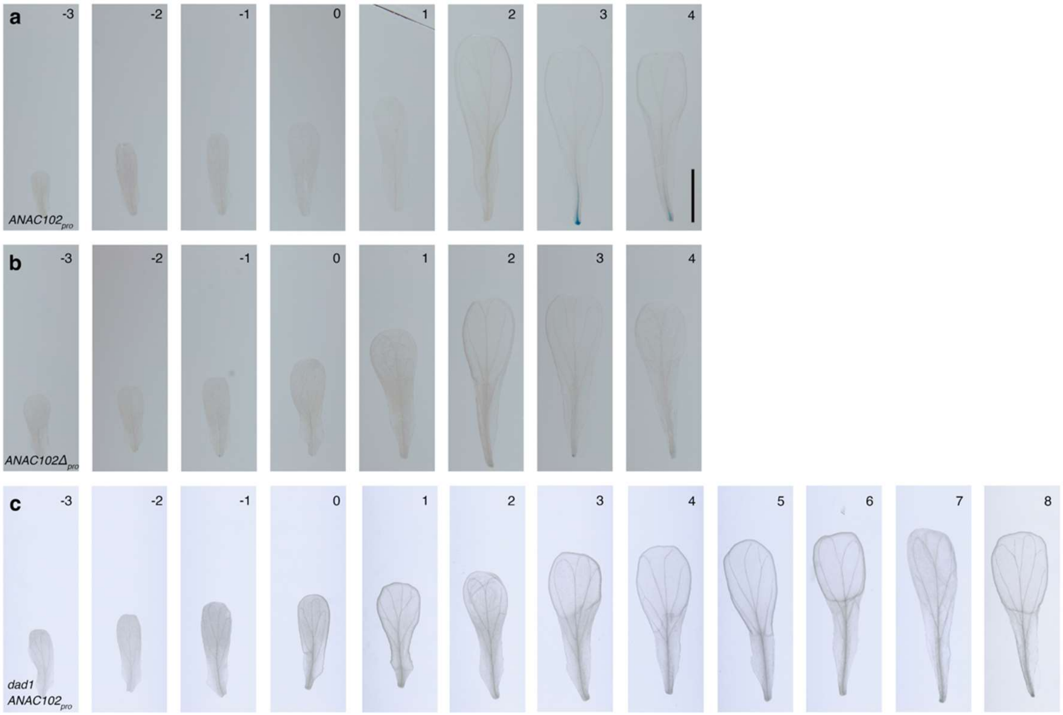

Supplementary Fig. 19 Entire petal images of *ANAC102*$_{pro}$*:GUS* reporter staining pattern during petal abscission.

a, **b**, GUS staining pattern for intact *ANAC102*$_{pro}$*:GUS* (a) and the G-box mutated variant *ANAC102Δ*$_{pro}$*:GUS* (b) reporter in petals from position –3 to +4 flowers. Scale bar = 1 mm. **c**, GUS staining pattern of the *ANAC102* promoter in *dad1* petals from position –3 to +8 flowers. Scale bar = 1 mm.

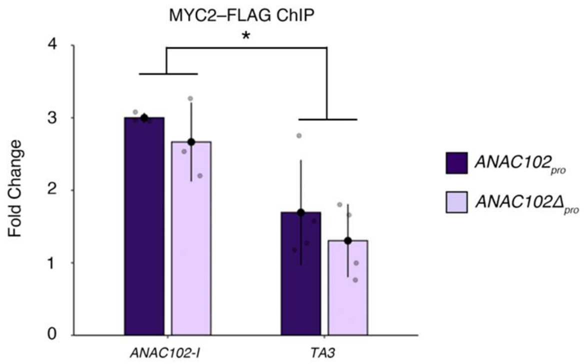

Supplementary Fig. 20 Binding of MYC2–FLAG to the endogenous *ANAC102* promoter.

Binding of MYC2–FLAG at the endogenous *ANAC102* promoter as shown by ChIP–qPCR. Position of PCR amplicons for ChIP–qPCR are shown in Fig. 5a. Data are means ± SEM. n = 4. Asterisks indicate significant differences of MYC2 binding between the endogenous *ANAC102* and *TA3* promoters based on two-tailed Student's t-test.

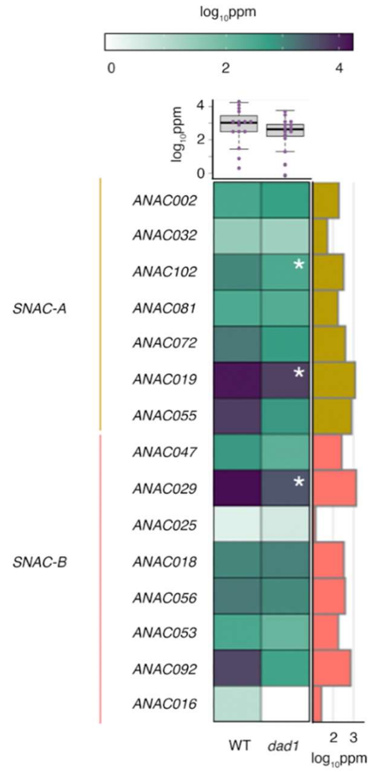

Supplementary Fig. 21 Expression of *SNAC* family genes in WT and the *dad1* mutant.
Relative expression levels of seven *SNAC-A* and eight *SNAC-B* genes in petals from WT and *dad1* plants from position +3 flowers. The heatmap shows the Log_{10} ppm based on RNA-seq data. The boxplot graph with individual data points is shown above the heatmap. The bar graph to the right shows expression levels of each gene in WT.

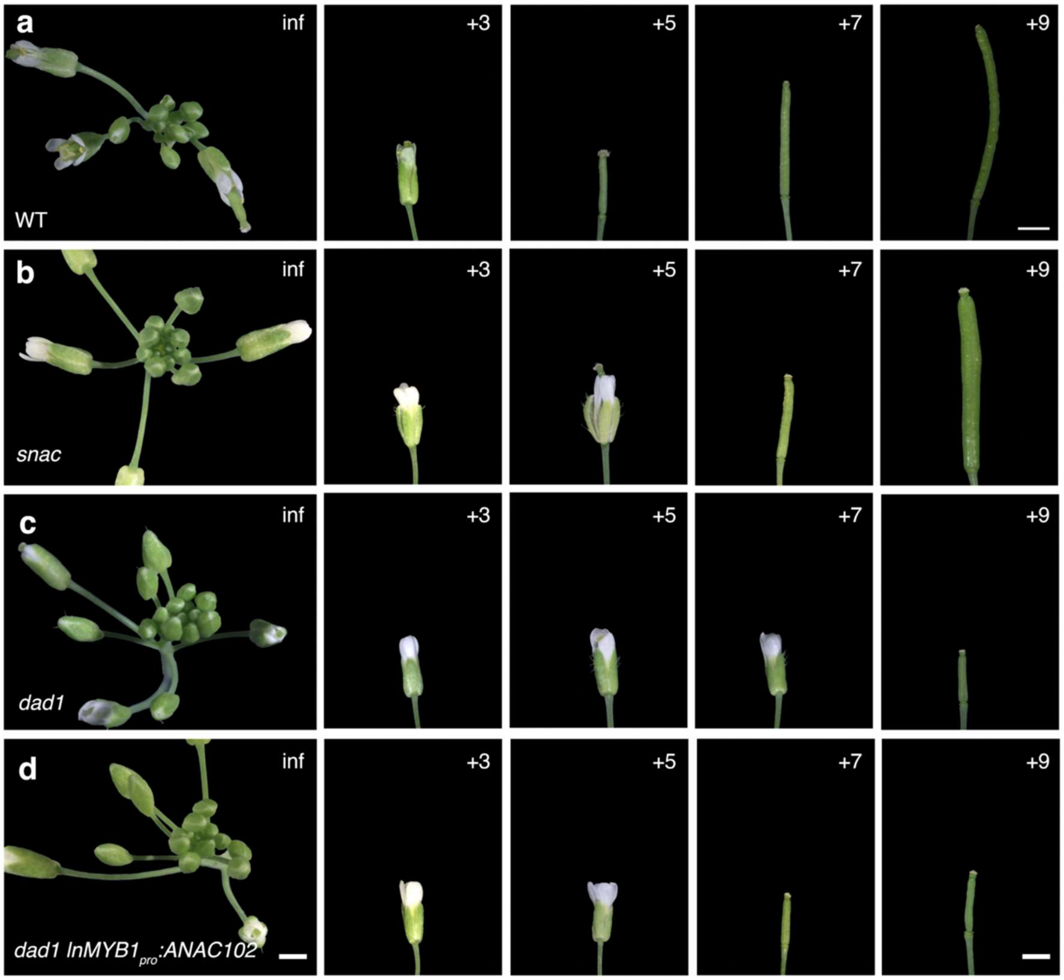

Supplementary Fig. 22 Close-up views of flowers from *snac* mutant and *ANAC102* misexpressor during petal abscission.

a-d, Left, Top views of WT, *snac*, *dad1*, and *dad1 InMYB1*$_{pro}$*:ANAC102* inflorescences. Right, Side views of WT, *snac*, *dad1*, and *dad1 InMYB1*$_{pro}$*:ANAC102* flowers at the indicated positions. Scale bar = 1 cm.

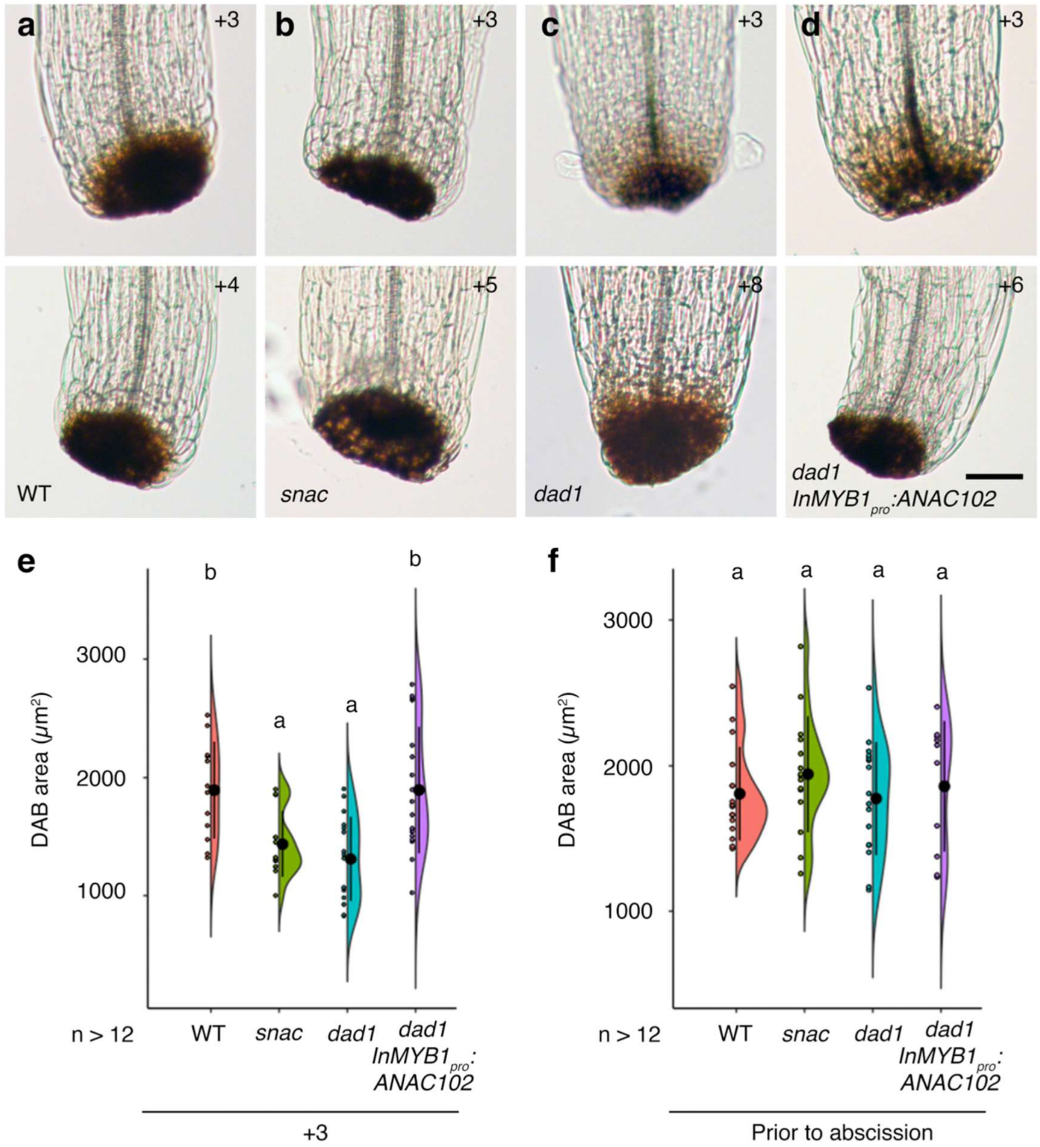

Supplementary Fig. 23 DAB staining in petals of flowers at two different positions during petal abscission.

a-d, DAB staining in petals of WT, *snac, dad1*, and *dad1 InMYB1*$_{pro}$*:ANAC102* flowers at position +3 (above) and just before petal abscission (below). Scale bar = 50 µm. **e, f,** Quantification of DAB-stained area (µm^2), shown as individual data points (left) and violin plots (right) for each genotype. Position +3 (c) and just before petal abscission (d) are shown. Black dots and vertical lines indicate mean and SD, respectively. $n > 12$. Different letters indicate significant differences, based on one-way ANOVA and post-hoc Tukey's HSD test ($p < 0.05$).

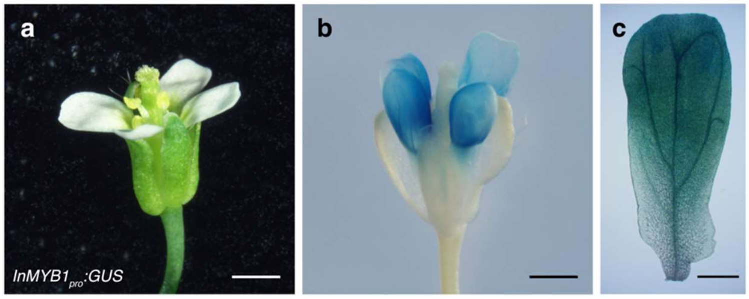

Supplementary Fig. 24 Petal-specific expression of *InMYB1* promoter.
a, Flower of the transgenic *InMYB1*$_{pro}$*:GUS* line. Scale bar = 1 mm. **b**, *InMYB1*$_{pro}$*:GUS* staining pattern in a wild-type flower at position +1. Scale bar = 1 mm. **c**, *InMYB1*$_{pro}$*:GUS* staining pattern in a WT petal from a position –1 flower. Scale bar = 200 µm.

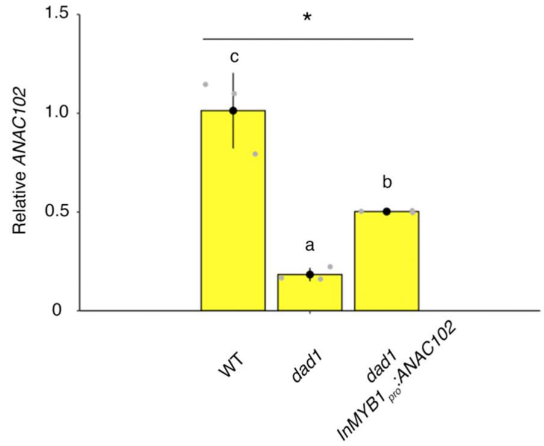

Supplementary Fig. 25 Expression levels of *ANAC102* in *InMYB1*$_{pro}$*:ANAC102* petals from position +3 flowers.

Relative *ANAC102* expression levels in the petals of WT, *dad1*, and *InMYB1*$_{pro}$*:ANAC102* flowers as determined by RT−qPCR. Data are means ± SEM. $n = 3$. Different letters indicate significant differences, based on one-way ANOVA and post-hoc Tukey's HSD test ($p < 0.05$).

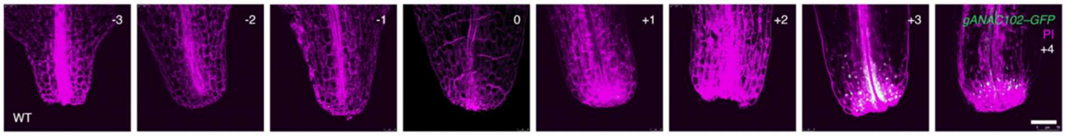

Supplementary Fig. 26 ANAC102–GFP accumulation in petal bases during petal abscission.

ANAC102−GFP accumulation in petal bases from position –3 to +4 flowers. Scale bar = 50 µm. Green, GFP; purple, propidium iodide (PI).

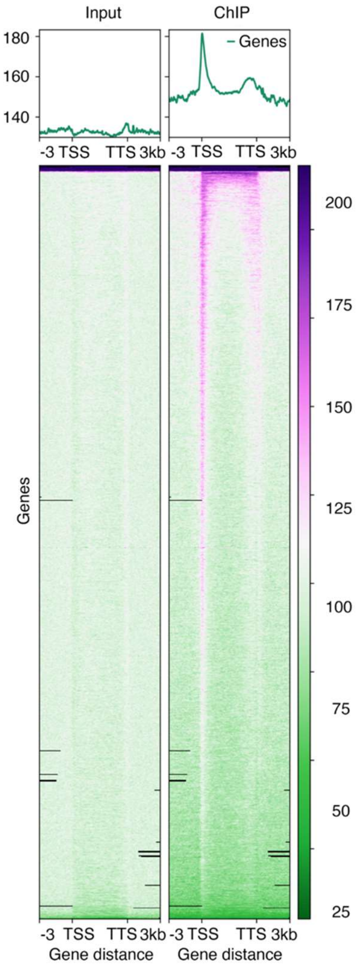

Supplementary Fig. 27 Positional profiles of ANAC102–GFP signal intensity on 4,196 ANAC102-bound genes.

Comparison between ANAC102 input and ChIP data. Left, Input; right, ChIP. The upper panels show the metaplot profile around each peak center. The lower panels show the density heatmaps over the gene body.

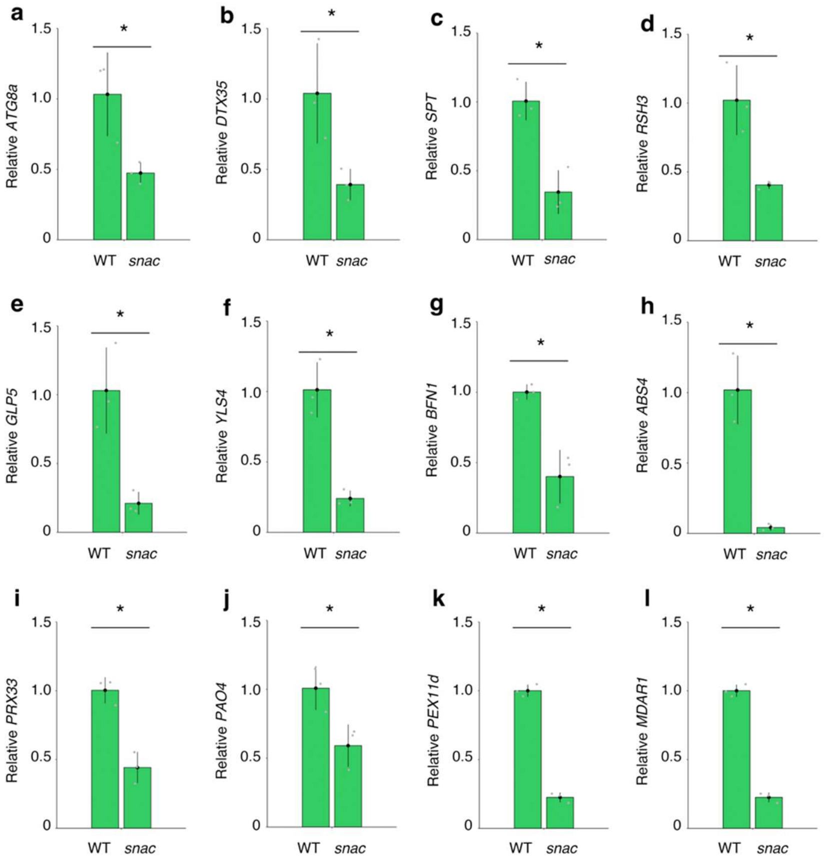

Supplementary Fig. 28 Expression levels of selected DEGs in WT and the *snac* mutant. **a-h**, Relative expression levels of the indicated genes in WT and the *snac* mutant by RT–qPCR. a, *ATG8a*. b, *DTX35*. c, *SPT*, d, *RSH3*. e, *GLP5*. f, *YLS4*. g, *BFN1*. h. *ABS4*. i, *PRX33*. j, *PAO4*. k, *PEX11d*. l, *MDAR1*. Data are means ± SEM. $n = 3$. Asterisks indicate significant differences between WT and the *snac* mutant based on two-tailed Student's *t*-test.

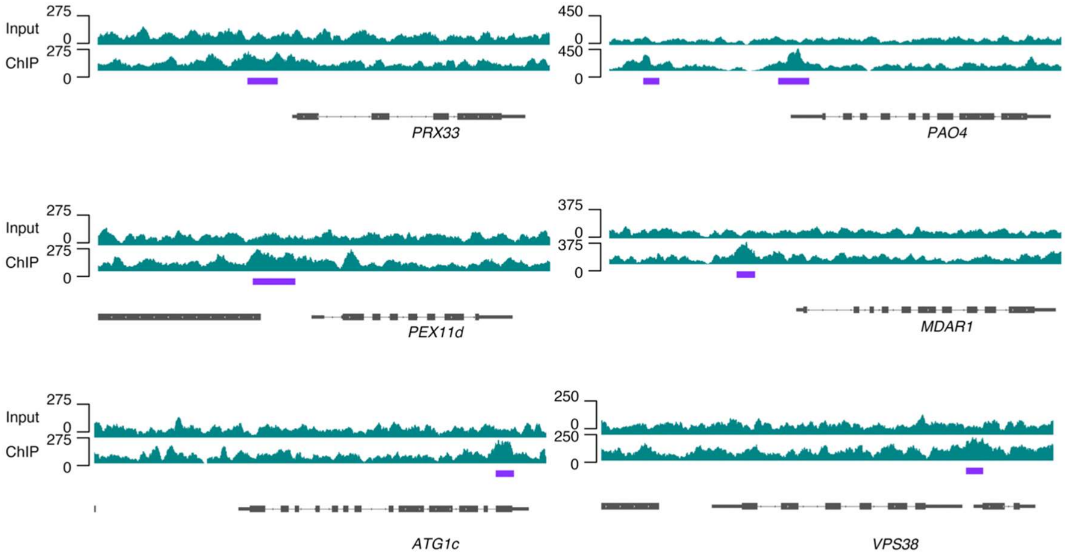

Supplementary Fig. 29 Binding peaks of ANAC102 at ROS- and autophagy-related loci. IGV browser view of ANAC102 input and ChIP signals. Purple horizontal bars indicate significant differences between ChIP and input signals. The gene models are shown as black bars and lines at the bottom of each panel.

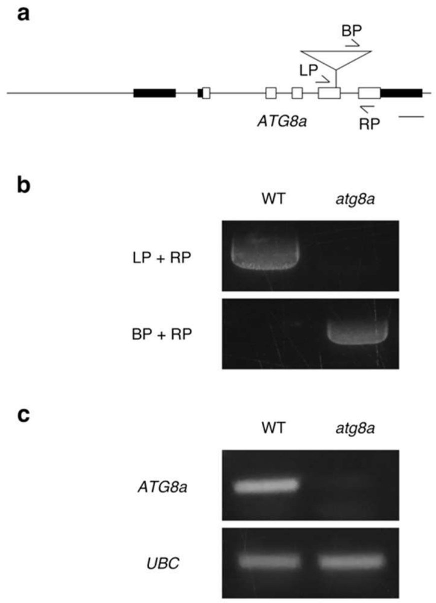

Supplementary Fig. 30 Genotyping of the *atg8a* mutant.
a, Diagram of the *ATG8a* locus and location of the T-DNA insertion. LP, left genomic primer; RP, right genomic primer; BP, T-DNA border primer. Scale bar, 100 bp. **b**, Genotyping PCR of WT and *atg8a* plants. The primers used for genotyping are shown above. **c**, Detection of *ATG8a* mRNA in WT and *atg8a*. *UBC* was used as a loading control.

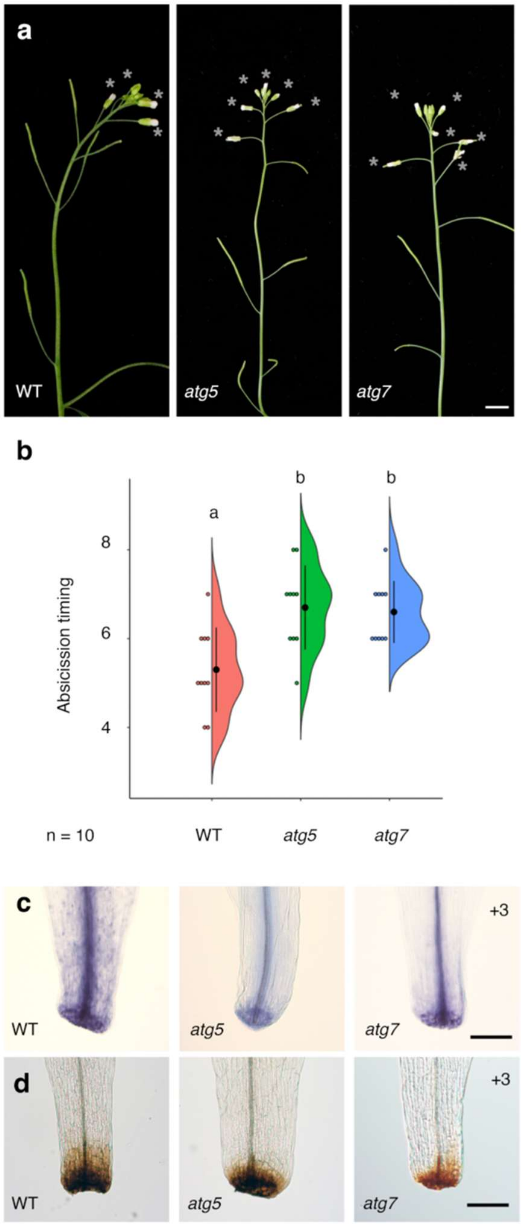

Supplementary Fig. 31 Petal abscission in the *atg5* and *atg7* mutants.
a, Profile views of WT, *atg5-1*, and *atg7-2* inflorescences. Open flowers with petals are indicated by asterisks. Scale bar = 1 cm. **b**, Quantification of the timing of abscission, shown as individual data points (left) and violin plots (right) for each genotype. Black dots and vertical lines indicate mean and standard deviation (SD), respectively. $n = 10$. Different letters indicate significant differences, based on one-way ANOVA and post-hoc Tukey's HSD test ($p < 0.05$). **c,** Trypan blue staining of WT, *atg5-1*, and *atg7-2* petals in position 3 flowers. Scale bar = 100 µm. **d,** DAB staining of WT, *atg5-1*, and *atg7-2* petals in position 3 flowers. Scale bar = 100 µm.

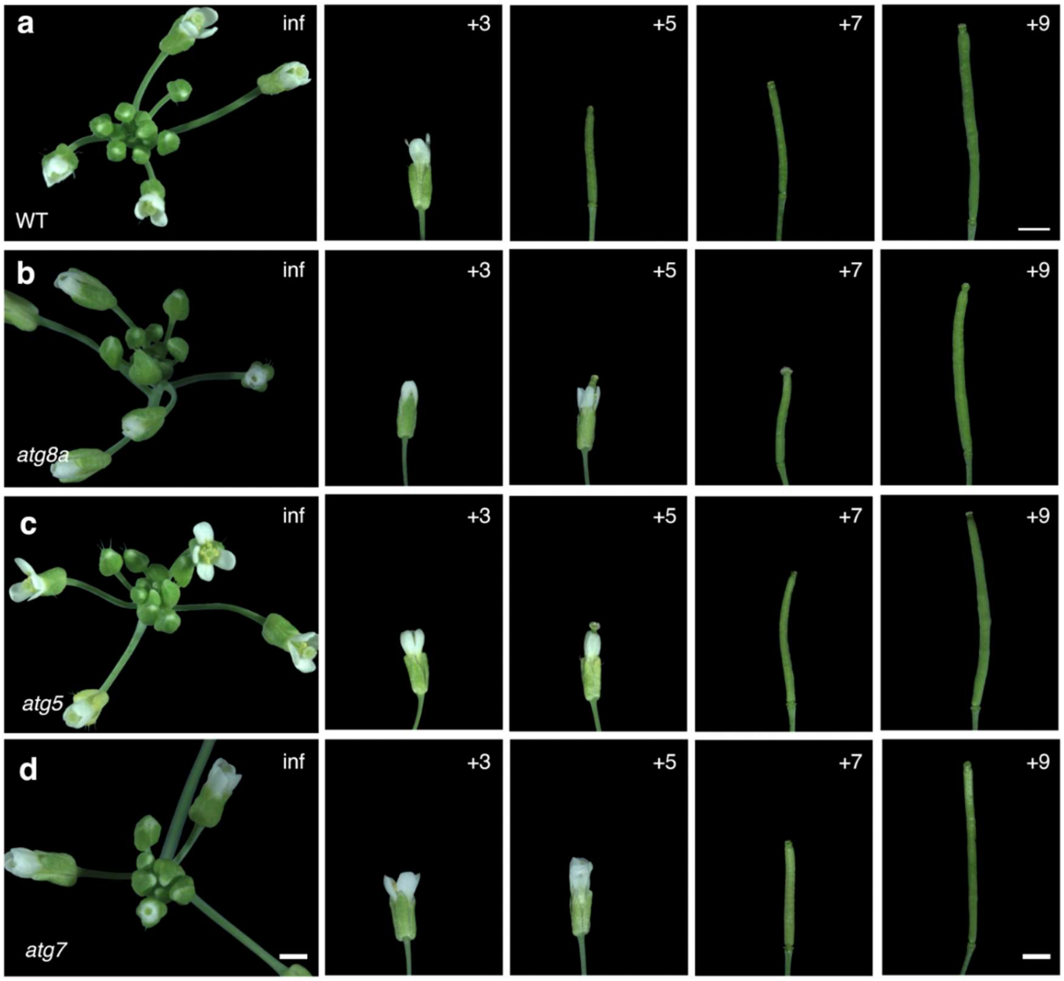

Supplementary Fig. 32 Close-up views of inflorescences and flowers from autophagy-related mutants during petal abscission.

a-d, Left, Top views of WT, *atg8a-1*, *atg5-1*, and *atg7-2* inflorescences. Right, Side views of WT, *atg8a-1*, *atg5-2*, and *atg7-2* flowers at the indicated positions. Scale bars, 1 cm.

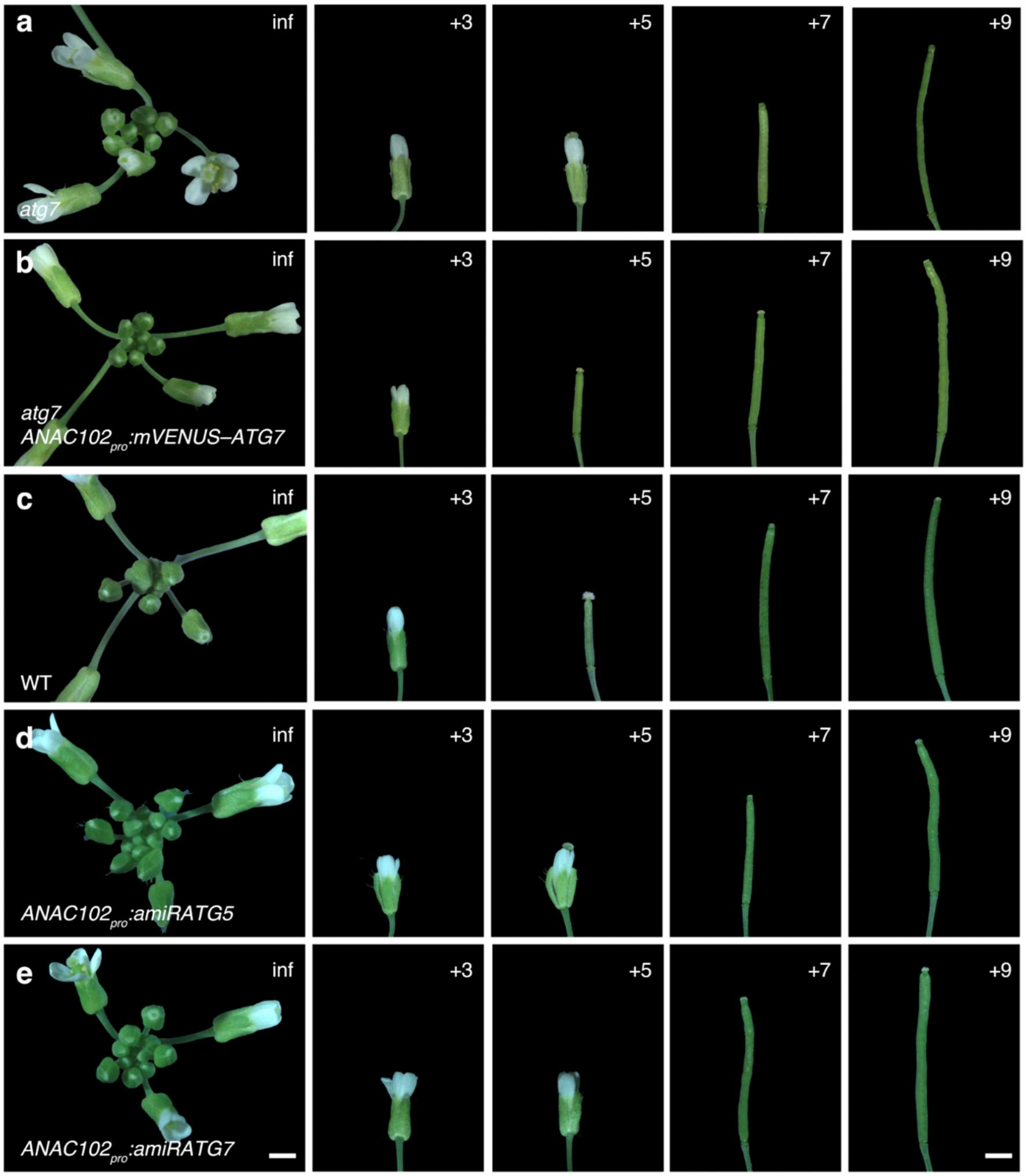

Supplementary Fig. 33 Close-up views of inflorescences and flowers from autophagy-related mutants and transgenic plants during petal abscission.

a-e, Left, Top views of *atg7-2*, *atg7-2 ANAC102*$_{pro}$*:mVENUS-ATG7,* WT, *ANAC102*$_{pro}$*:amiRATG5*, and *ANAC102*$_{pro}$*:amiRATG7*. Right, Side views of *atg7-2*, *atg7-2 ANAC102*$_{pro}$*:mVENUS-ATG7,* WT, *ANAC102*$_{pro}$*:amiRATG5*, and *ANAC102*$_{pro}$*:amiRATG7* flowers at the indicated positions. Scale bar = 1 cm.

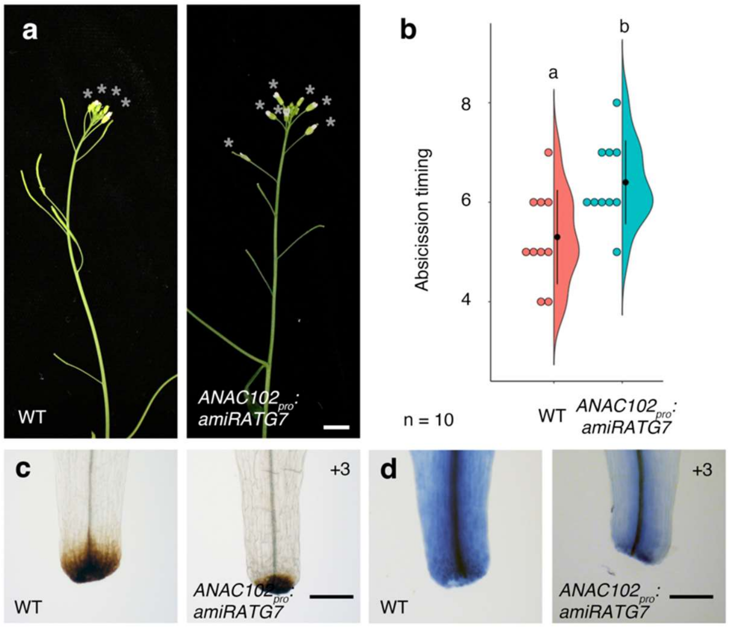

Supplementary Fig. 34 Petal abscission in *ANAC102$_{pro}$:amiRATG7* plants.
a, Profile views of WT and *ANAC102$_{pro}$:amiRATG7* inflorescences. Open flowers with petals are indicated by asterisks. Scale bar = 1 cm. **b**, Quantification of the timing of abscission, shown as individual data points (left) and violin plots (right) for each genotype. Black dots and vertical lines indicate mean and standard deviation (SD), respectively. n = 10. Different letters indicate significant differences based on one-way ANOVA and post-hoc Tukey's HSD test (p < 0.05). **c,** Trypan blue staining of WT and *ANAC102$_{pro}$:amiRATG7* petals in position +3 flowers. Scale bar = 100 µm. **d,** DAB staining of WT and *ANAC102$_{pro}$:amiRATG7* petals in position +3 flowers. Scale bar = 100 µm.

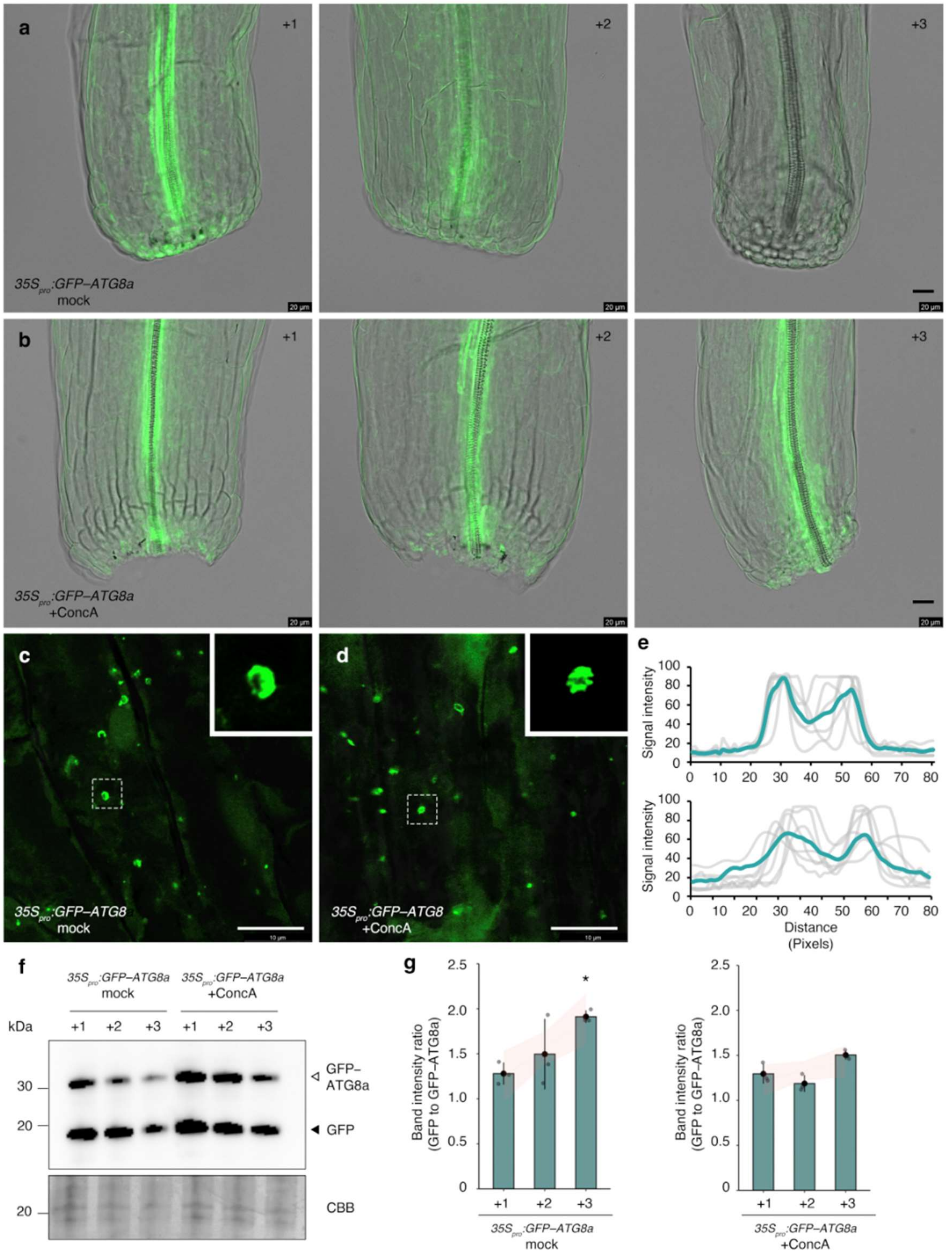

Supplementary Fig. 35 Autophagosome degradation at the petal base is delayed in concanamycin A-treated plants during petal abscission.

a, **b**, Spatiotemporal observation of mock- (a) and concanamycin A-treated (b) *35S$_{pro}$:GFP–ATG8a* petal bases by confocal microscopy. Numbers in the right corners indicate flower positions. Scale bars = 20 µm. **c, d**, Higher magnification images of mock- (c) and concanamycin A-treated (d) *35S$_{pro}$:GFP–ATG8a* petal bases by confocal microscopy. Images in insets are magnified images of the boxed regions. The punctate structures labeled by green fluorescence from the cleavage of GFP–ATG8a indicate autophagosome-related

structures. Scale bars = 10 µm. **e**, GFP-ATG8a signal intensity profile in mock- and concanamycin A-treated $35S_{pro}$:*GFP–ATG8a* autophagosome-like structures. $n > 8$. Each gray line indicates an individual trace. The green line shows average data. **f**, Immunoblot analysis showing the processing of *GFP–ATG8a* after mock and concanamycin A treatment during petal abscission. Crude protein extracts from petals at the indicated positions were subjected to SDS-PAGE and immunoblot analysis with anti-GFP antibodies. Arrows and arrowheads to the right of the immunoblot image indicate GFP–ATG8a and free GFP, respectively. Coomassie Brilliant Blue (CBB) staining served as a loading control. **g**, Quantification of the GFP/ GFP–ATG8a ratio during petal abscission based on densitometric scans of the immunoblots shown in g. Values are means ± SEM. $n = 5$. Asterisks indicate significant differences between mock- and concanamycin A-treated $35S_{pro}$:*GFP–ATG8a* plants based on two-tailed Student's *t*-test.
