# Supplementary material for: Petal abscission is promoted by jasmonic acid-induced autophagy at Arabidopsis petal bases
Source: Nat Commun. 2024 Feb 6;15:1098. doi: 10.1038/s41467-024-45371-3 (PMC10847506; doi:10.1038/s41467-024-45371-3)
Supplement: Supplementary file 3 — Description of Additional Supplementary Files [file 41467_2024_45371_MOESM3_ESM.pdf]

Description of Additional Supplementary Files

Supplementary Data 1. Summary of RNA-seq data in wild-type and *dad1* petals from position 3 flowers.

Supplementary Data 2. List of 779 differentially expressed genes between the wild type and the *dad1* mutant during petal abscission.

Supplementary Data 3. List of 70 high-confidence genes regulated by jasmonic acid during petal abscission.

Supplementary Data 4. GO terms for the 70 high-confidence genes regulated by jasmonic acid during petal abscission.

Supplementary Data 5. *Cis*-elements identified using the promoter sequences of 70 high-confidence genes.

Supplementary Data 6. ChIP-seq data obtained from *gANAC102–GFP* transgenic plants.

Supplementary Data 7. *Cis*-element identification using *ANAC102–GFP* ChIP-seq.

Supplementary Data 8. Summary of RNA-seq data from the wild type and *snac* mutant from position +3 flowers.

Supplementary Data 9. List of 2,615 differentially expressed genes between the wild type and the *snac* mutant during petal abscission.

Supplementary Data 10. List of GO terms for 415 genes directly activated by ANAC102 during petal abscission.

Supplementary Data 11. List of 581 genes regulated by DAD1 and ANAC102 during petal abscission.

Supplementary Data 12. Primers used in this study.
