# Supplementary material for: Petal abscission is promoted by jasmonic acid-induced autophagy at Arabidopsis petal bases
Source: Nat Commun. 2024 Feb 6;15:1098. doi: 10.1038/s41467-024-45371-3 (PMC10847506; doi:10.1038/s41467-024-45371-3)
Supplement: Supplementary file 16 — Reporting Summary [file 41467_2024_45371_MOESM16_ESM.pdf]

nature portfolio

Corresponding author(s): Toshiro Ito and Nobutoshi Yamaguchi

Last updated by author(s): Jan 3, 2024

Reporting Summary

Nature Portfolio wishes to improve the reproducibility of the work that we publish. This form provides structure for consistency and transparency in reporting. For further information on Nature Portfolio policies, see our Editorial Policies and the Editorial Policy Checklist.

Statistics

For all statistical analyses, confirm that the following items are present in the figure legend, table legend, main text, or Methods section.

n/a	Confirmed	
☐	☒	The exact sample size (*n*) for each experimental group/condition, given as a discrete number and unit of measurement
☐	☒	A statement on whether measurements were taken from distinct samples or whether the same sample was measured repeatedly
☐	☒	The statistical test(s) used AND whether they are one- or two-sided<br>*Only common tests should be described solely by name; describe more complex techniques in the Methods section.*
☒	☐	A description of all covariates tested
☐	☒	A description of any assumptions or corrections, such as tests of normality and adjustment for multiple comparisons
☐	☒	A full description of the statistical parameters including central tendency (e.g. means) or other basic estimates (e.g. regression coefficient) AND variation (e.g. standard deviation) or associated estimates of uncertainty (e.g. confidence intervals)
☐	☒	For null hypothesis testing, the test statistic (e.g. *F*, *t*, *r*) with confidence intervals, effect sizes, degrees of freedom and *P* value noted<br>*Give P values as exact values whenever suitable.*
☒	☐	For Bayesian analysis, information on the choice of priors and Markov chain Monte Carlo settings
☒	☐	For hierarchical and complex designs, identification of the appropriate level for tests and full reporting of outcomes
☒	☐	Estimates of effect sizes (e.g. Cohen's *d*, Pearson's *r*), indicating how they were calculated

Our web collection on statistics for biologists contains articles on many of the points above.

Software and code

Policy information about availability of computer code

Data collection	ChIP-seq data were collected from the National Genomics Data Center or Gene Expression Omnibus (MYC2/MED: CRA001078 [https://ngdc.cncb.ac.cn/gsa/browse/CRA001078]; H3K9ac: GSE67776 [https://www.ncbi.nlm.nih.gov/geo/query/acc.cgi?acc=GSE67776], GSE67777 [https://0-www-ncbi-nlm-nih-gov.brum.beds.ac.uk/geo/query/acc.cgi?acc=GSE67777], and GSE67778 [https://0-www-ncbi-nlm-nih-gov.brum.beds.ac.uk/geo/query/acc.cgi?acc=GSE67778]; nucleosome occupancy: GSE50242 [https://www.ncbi.nlm.nih.gov/geo/query/acc.cgi?acc=gse50242]; DNase I-hypersensitive site: GSE34318 [https://www.ncbi.nlm.nih.gov/geo/query/acc.cgi?acc=GSE34318]).Flower-specific expression data was obtained from the TraVA website (http://travadb.org/browse/). All databases associated with the software and packages used in the study are described in the "Methods" section. Source data are provided with this paper.
Data analysis	All software used (including the version and parameter, when not default) is indicated in the Methods section.

For manuscripts utilizing custom algorithms or software that are central to the research but not yet described in published literature, software must be made available to editors and reviewers. We strongly encourage code deposition in a community repository (e.g. GitHub). See the Nature Portfolio guidelines for submitting code & software for further information.

Data

Policy information about availability of data

All manuscripts must include a data availability statement. This statement should provide the following information, where applicable:

- Accession codes, unique identifiers, or web links for publicly available datasets
- A description of any restrictions on data availability
- For clinical datasets or third party data, please ensure that the statement adheres to our policy

The RNA-seq and ChIP-seq data have been deposited in the DDBJ database (DRA014836 [https://ddbj.nig.ac.jp/resource/sra-submission/DRA014836], DRA014637

[https://ddbj.nig.ac.jp/resource/sra-submission/DRA014637]). Main source codes that are used in this work are available on the GitHub repository [https://zefeng2018.github.io/Plant-Gene-Expression-Prediction/]. The plant materials are available from corresponding authors upon request.

Field-specific reporting

Please select the one below that is the best fit for your research. If you are not sure, read the appropriate sections before making your selection.

- [x] Life sciences
- [ ] Behavioural & social sciences
- [ ] Ecological, evolutionary & environmental sciences

For a reference copy of the document with all sections, see nature.com/documents/nr-reporting-summary-flat.pdf

Life sciences study design

All studies must disclose on these points even when the disclosure is negative.

Sample size	Sample sizes were determined based on prior experience and typical standards in the field. Rafael Rodrigues de Souza, Marcos Toebe, Volmir Sergio Marchioro, Alberto Cargnelutti Filho, Karina Chertok Bittencourt, Anderson Chuquel Mello, João Antônio Paraginski, Sample size and modeling of plant variability using precision statistics in soybean counting traits, Field Crops Research, Volume 291, 2023, 108789, ISSN 0378-4290, https://doi.org/10.1016/j.fcr.2022.108789. (https://www.sciencedirect.com/science/article/pii/S0378429022003604) No statistical method was used to predetermine sample size. For statistical tests (i.e. Chi-Square, Student's t-test), at least 3 biological replicates were included to ensure enough sample size.
Data exclusions	Only experimental group and control group were included in the analyses. Any plants without these traits were excluded by phenotyping and/or genotyping.
Replication	All experiments were repeated at least three times, and attempts at replication were successful.
Randomization	Random selection was not conducted. Plants in either experimental group or control group were included in the study. These two groups were distinct from each other; experimental group was compared with control group for phenotyping/expression.
Blinding	Blinding was not applied since experimental group and control group were distinct from each other.

Reporting for specific materials, systems and methods

We require information from authors about some types of materials, experimental systems and methods used in many studies. Here, indicate whether each material, system or method listed is relevant to your study. If you are not sure if a list item applies to your research, read the appropriate section before selecting a response.

Materials & experimental systems

n/a	Involved in the study	
	x	Antibodies
x		Eukaryotic cell lines
x		Palaeontology and archaeology
x		Animals and other organisms
x		Human research participants
x		Clinical data
x		Dual use research of concern

Methods

n/a	Involved in the study	
	x	ChIP-seq
x		Flow cytometry
x		MRI-based neuroimaging

Antibodies

Antibodies used	anti-GFP antibody (SAB4301138, Sigma): 1:200 for ChIP<br>anti-MYC antibody (9B11, Cell Signaling): 1:1000 for ChIP<br>anti-FLAG M2 antibody (F3165, Sigma-Aldrich): 1:1000 for ChIP<br>anti-H3K9ac antibody (31-1054-00, ReMAb Bioscience) : 1:1000 for ChIP<br>anti-Pol II antibody (ab817, abcam) : 1:1000 for ChIP<br><br>Rabbit polyclonal anti-GFP (ab290, Abcam): 1:100 for In situ PLA<br>rabbit anti-HA (12CA5, Roche): 1:100 for In situ PLA<br>anti-FLAG M2 antibody (F3165, Sigma-Aldrich): 1:100 for In situ PLA<br><br>Rabbit polyclonal anti-GFP (ab290, Abcam): 1:1000 for WB<br>anti-H3 (ab1791, Abcam) : 1:1000 for WB<br>anti-rabbit IgG HRP conjugate (31460, Thermo Fisher Scientific): 1:5000 for ChIP
Validation	The antibodies were validated by the suppliers.

https://www.sigmaaldrich.com/JP/ja/product/sigma/sab4301138
https://www.cellsignal.com/products/primary-antibodies/myc-tag-9b11-mouse-mab/2276
https://www.revmab.com/index.php/product-tag/31-1054-00/
https://www.abcam.com/rna-polymerase-ii-ctd-repeat-ysptsps-antibody-8wg16-chip-grade-ab817.html
https://www.abcam.com/gfp-antibody-ab290.html
https://www.thermofisher.com/antibody/product/Goat-anti-Rabbit-IgG-H-L-Secondary-Antibody-Polyclonal/31460
https://www.abcam.co.jp/histone-h3-antibody-nuclear-marker-and-chip-grade-ab1791.html
https://www.sigmaaldrich.com/PH/en/product/roche/roaha

ChIP-seq

Data deposition

☒ Confirm that both raw and final processed data have been deposited in a public database such as GEO.

☒ Confirm that you have deposited or provided access to graph files (e.g. BED files) for the called peaks.

Data access links<br>*May remain private before publication.*	The data was uploaded onto DDBJ website (DRA014637 [https://ddbj.nig.ac.jp/resource/sra-submission/DRA014637]).
Files in database submission	*Provide a list of all files available in the database submission.*
Genome browser session<br>(e.g. UCSC)	*Provide a link to an anonymized genome browser session for "Initial submission" and "Revised version" documents only, to enable peer review. Write "no longer applicable" for "Final submission" documents.*

Methodology

Replicates	Replicates agreed well. Data was further confirmed by ChIP-qPCR
Sequencing depth	Sample Name Total reads Trimmed Reads Mapped reads Mapping rate<br>NAC102_ChIP 29265224 28209622 25709019 91.14%<br>NAC102_Input 29838946 28715335 26113306 90.94%
Antibodies	anti-GFP antibody (SAB4301138, Sigma) 1:200 for ChIP
Peak calling parameters	Main source codes that are used in this work are available on the GitHub repository [https://zefeng2018.github.io/Plant-Gene-Expression-Prediction/]. Peak calling is described in the methods.
Data quality	Main source codes that are used in this work are available on the GitHub repository [https://zefeng2018.github.io/Plant-Gene-Expression-Prediction/]. Peak attributes are described in the methods.
Software	Software used is described in the methods.
